# Cerebrospinal Fluid Biomarkers and Prediction of Conversion in Patients with Mild Cognitive Impairment: 4-Year Follow-Up in a Routine Clinical Setting

**DOI:** 10.1100/tsw.2009.106

**Published:** 2009-09-15

**Authors:** Alessia Lanari, Lucilla Parnetti

**Affiliations:** ^1^ Neurology Department, Mantova General Hospital, Italy; ^2^ Centre for Memory Disturbances, Section of Neurology, University of Perugia, Italy

**Keywords:** CSF biomarkers, mild cognitive impairment, Alzheimer’s disease, dementia

## Abstract

Mild cognitive impairment (MCI) is a very common syndrome in elderly people, with a high risk of conversion to dementia. Several investigations have shown the usefulness of cerebrospinal fluid (CSF) biomarkers (Aβ42, total tau [T-tau], and phosphorylated tau [P-tau]) in predicting the progression to Alzheimer's disease (AD). We report a 4-year follow-up of MCI patients who underwent CSF evaluation for biomarker assessment, in order to further evaluate the usefulness of CSF analysis in predicting the conversion to dementia in a routine clinical setting. We identified 55 patients with MCI among the consecutive patients, referred from 2001 to 2003 to our Memory Clinic for cognitive disorders, who underwent a complete diagnostic assessment, including lumbar puncture (n = 273). At the end of the follow-up, 31 MCI patients (56%) did not progress to dementia (stable MCI), while 24 (44%) developed a dementia condition. At baseline, the mean levels of CSF Aβ42, T-tau, and P-tau were significantly altered in MCI patients who were converting to dementia with respect to those with stable MCI. All MCI patients with the three altered CSF biomarkers developed dementia within 1 year. Among the stable MCI patients, none showed all three pathological values and only one subject had the pathological value of P-tau. Early diagnosis of dementia and, specifically, a correct prediction of MCI outcome represent a primary goal. To this respect, the role of CSF biomarkers seems to be crucial in a routine clinical setting.

## INTRODUCTION

Mild cognitive impairment (MCI) is an etiologically heterogeneous syndrome characterized by cognitive impairment shown by objective measures adjusted for age and education in advance of dementia; approximately 12% of MCI patients convert to Alzheimer's disease (AD) or other dementia disorders every year[1]. The degenerative process in the AD brain starts several years before the clinical onset of the disease[2,3]. During this preclinical period, there is a gradual loss of axons and neurons, and at a certain threshold, the first symptoms appear. At this stage, patients do not fulfill the criteria for dementia and may be diagnosed with MCI. However, MCI is a very common syndrome in elderly people and a multitude of causes are recognized; therefore, apart from the subgroup that evolves to AD, many other MCI cases may not evolve to any dementia or may develop non-AD dementias[4].

Biomarkers can aid in the prediction of progression to AD in individuals with MCI. Such methods would be of even greater significance if new, disease-modifying, drug candidates, such as β-sheet breakers, β-secretase inhibitors, and $\beta_{1\text{-}42}$ (Aβ42) immunotherapy, are applied. These therapeutic interventions are likely to have the best efficacy in the early or even prodromal phase of the disease, when the synaptic and neuronal loss has not become too widespread[5]. In AD patients, cerebrospinal fluid (CSF) $A\beta_{42}$ concentrations are consistently decreased by about 50% with respect to controls. This decrease has been associated with enhanced deposition of $A\beta_{42}$ in the brain[6]. CSF total tau (T-tau) is, on average, increased two- to threefold in AD, as well as phosphorylated tau (P-tau)[7]. Pathological values in two or more CSF biomarkers reliably predict MCI conversion to AD and correctly identify the stable form of MCI[5,8,9,10]. However, the clinical follow-up in these studies has been relatively short, generally 1–2 years. Hansson and colleagues recently confirmed, with a follow-up study of about 6 years, that the association between pathological CSF and progression to AD in MCI patients was strong and independent of established risk factors, including age, sex, education, APO genotype, and plasma homocysteine[11].

Here we report a 4-year follow-up of MCI patients who underwent CSF evaluation for biomarker assessment, in order to further evaluate the usefulness of CSF analysis in a routine clinical setting.

## MATERIALS AND METHODS

### Subjects

In the period from January 2001 to June 2003, 273 patients underwent a thorough screening for cognitive decline at our Memory Clinic; 75% of them were referred by general practitioners. Fifty-five partients fulfilled the criteria for MCI. Neuropsychological evaluation included the Mini-Mental State Examination (MMSE), the Milan Overall Dementia Assessment (MODA), the Clinical Dementia Rating (CDR), assessment of advanced and basic activities of daily living, and the Neuropsychiatric Inventory (NPI).

Patients with other causes of cognitive impairment, including subcortical vascular dementia, metabolic diseases, alcohol abuse, brain tumor, subdural hematoma, and CNS infection (n = 94), were excluded. Patients with no secondary causes of cognitive impairment at the first screening underwent lumbar puncture (LP) for CSF biomarker determination. LPs were performed after informed consent had been obtained. CSF (10 ml) was collected in sterile polypropylene tubes. In the native CSF, determination of routine chemical parameters (leukocyte and erythrocyte cell count, glucose, lactate, total protein content, IgG index, TPHA) was performed.

The criteria used for MCI were those defined by Petersen and collaborators[1], and included memory complaint, preferably corroborated by an informant; objective memory impairment, adjusted for age and education; preservation of general cognitive functioning; and no or minimum impairment of daily life activities.

Patients with MCI were regularly followed up by experienced physicians, specialized in cognitive disorders, for at least 4 years. MCI patients underwent a complete clinical and neuropsychological evaluation every 6th month. The diagnosis of AD was done according to the NINCDS-ADRDA criteria for probable AD[12]; frontotemporal dementia (FTD) was defined according to the Lund-Manchester Consensus criteria[13], dementia with Lewy bodies (DLB) was diagnosed in line with the criteria stated by McKeith et al.[14], and progressive supranuclear palsy (PSP) was defined according to criteria stated by Litvan et al.[15].

## CSF Measurements

The LPs were carried out in the morning between 8:00 and 10:00 a.m., in a sitting position, after an overnight rest, with the patient fasting for 12 h. Ten ml of CSF was withdrawn in polypropylene tubes, then centrifuged for 10 min at 4000 ×*g*, and 500-µl aliquots in Eppendorf tubes were frozen at –80°C until analysis. CSF biomarkers were determined by ELISA method (Innotest hTAU-Ag, Innotest pTAU181-Ag, Innotest β-amyloid 1–42, Innogenetics NV, Gent, Belgium). According to Sjögren et al.[16], the cutoff for CSF-Aβ42 protein, independent of age, was fixed at 500 pg/ml for healthy subjects; for T-tau protein, a value ≤300 pg/ml was considered normal for subjects 21–50 years old, ≤450 pg/ml for subjects between 51 and 70 years of age, and <500 pg/ml for persons older than 71 years[16]. For P-tau181, we used the cutoff <80 pg/ml[8].

## RESULTS

Among the consecutive patients referred to our Memory Clinic for cognitive disorders who underwent a complete diagnostic assessment (n = 273), we identified 55 patients with MCI. In Table 1, the main clinical and biochemical details at baseline of the patients studied are reported.

**TABLE 1**
**Values of CSF Biomarkers in All Groups at Baseline (Mean ± SD; Median, Range)**

| | Age | MMSE | Aβ24 (pg/ml) | T-tau (pg/ml) | P-tau (pg/ml) |
|---|---|---|---|---|---|
| MCI (n = 55) | 67 ± 8 | 27 ± 1 | 623 ± 277<br>585 (200–1190) | 422 ± 286<br>285 (75–1190) | 65 ± 32<br>58 (25–148) |
| AD (n = 100) | 68 ± 9 | 15 ± 3 | 411 ± 148<br>395 (130–790) | 628 ± 314<br>575 (80–1300) | 136 ± 59<br>125 (60–326) |
| FTD (n = 11) | 61 ± 9 | 19 ± 4 | 441 ± 141<br>430 (190–680) | 529 ± 374<br>435 (90–1000) | 65 ± 35<br>(22–122) |
| DLB (n = 14) | 68 ± 8 | 12 ± 3 | 386 ± 142<br>385 (210–592) | 458 ± 397<br>270 (60–700) | 39 ± 21<br>32 (15–88) |

## Clinical Outcome

At the end of follow-up, 31 (56%) patients did not progress to dementia (stable MCI), while 24 (44%) developed a dementia condition (converter MCI). As shown in Fig. 1, 11/24 (46%) of converters developed dementia in the first year, 8/24 (33.3%) in the second year, 3/24 (12.5%) in the third year, and 2/24 (8.3%) in the fourth year of follow-up. In Fig. 2, the clinical evolution observed in our MCI group is reported. It can be seen that 18/24 developed AD and 6 progressed to other forms of dementia (n = 3 FTD, n = 1 DLB, n = 2 PSP).

## CSF Biomarkers

At baseline (Table 2), CSF Aβ42 was significantly lower in MCI patients converting to dementia than in stable MCI patients ($p < 0.0001$). Accordingly, both T-tau and P-tau were significantly higher in MCI converting to dementia than in stable MCI ($p < 0.018$ and $p < 0.001$, respectively). About stable MCI patients, it is noteworthy that only one patient had the pathological value of P-tau, and 93% of them showed either none or one pathological value of biomarkers; none of them showed all three pathological

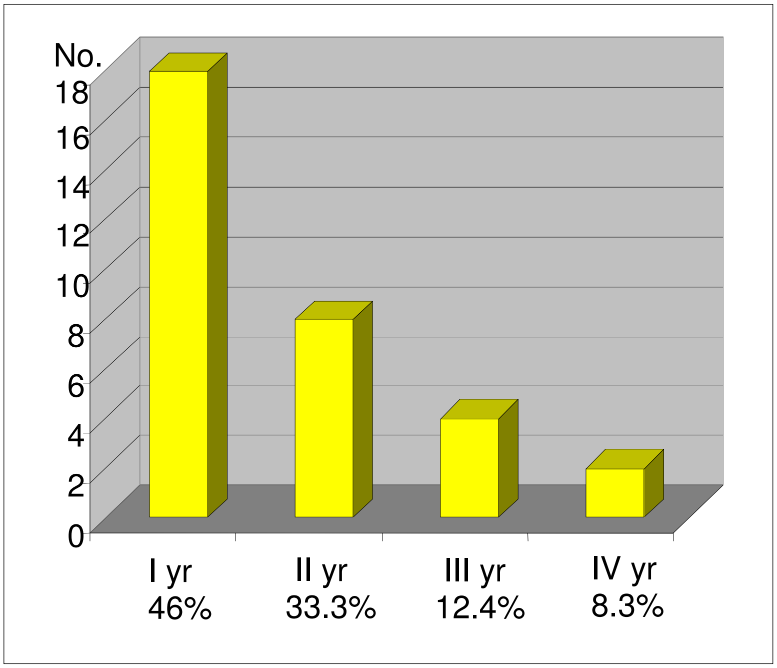

**FIGURE 1.** Conversion rate per year in the MCI group.

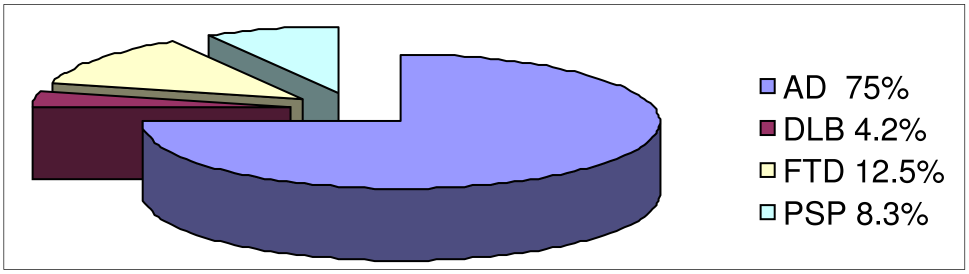

**FIGURE 2**. Clinical evolution observed in the MCI patients converting to dementia.

values. In Table 3, CSF biomarker values obtained in the MCI group converting to AD (MCI-AD) and in the MCI group converting to other forms of dementia (MCI-OD) are reported.

At the end of the follow-up, 14 (60%) of 24 converter patients showed two or more biomarkers with pathological values. In particular, among MCI patients who converted to AD, five showed all three pathological values, while nobody disclosed normal values. All MCI patients with the three altered CSF biomarkers developed dementia within 1 year.

## DISCUSSION

A correct prediction of MCI outcome represents a primary goal in the diagnosis of dementia; to this respect, the role of CSF biomarkers seems to be crucial in a routine clinical setting. In this context, our results confirm this concept. In fact, all MCI patients who progressed to AD in the 4 years had pathological values of CSF biomarkers at baseline, further confirming previous reports[5,9,11,17]. In particular, the finding that all patients with the three pathological values developed dementia within the

**TABLE 2**
**Values of CSF Biomarkers (Mean ± S.D.) in Stable and Converter MCI Subgroups**

| | Aβ24 (pg/ml) | T-tau (pg/ml) | P-tau (pg/ml) |
|---|---|---|---|
| Stable MCI (n = 31) | 942 ± 490 | 235 ± 230 | 40 ± 12 |
| Converter MCI (n = 24) | 403 ± 193** | 430 ± 304* | 90 ± 30** |

* $p < 0.01$.

** $p < 0.001$.

**TABLE 3**
**Values of CSF Biomarkers (Mean ± S.D.) in MCI Group Converting to AD (MCI-AD) and MCI Converting to Other Dementias (MCI-OD*)**

| | Aβ24 (pg/ml) | T-tau (pg/ml) | P-tau (pg/ml) |
|---|---|---|---|
| Converter MCI-AD (n = 18) | 402 ± 195 | 402 ± 318 | 91 ± 31 |
| Converter MCI-OD (n = 6) | 445 ± 194 | 457 ± 287 | 83 ± 28 |

* DLB, n = 1; FTD, n = 3; PSP, n = 2.

first year of follow-up is quite important, which is strong evidence of the practical value of these biomarkers in the single MCI case.

Our survey also shows the importance of CSF biomarkers when defining a "benign" (i.e., stable) MCI, since only one case showed an increase of P-tau values, and none had all three pathological values.

When considering sensitivity, specificity, reliability, costs, and invasiveness of different markers proposed (neuropsychology, structural/functional neuroimaging, CSF biomarkers), we believe that CSF biomarkers are a first-line choice; also when selecting very early AD cases to be treated with the new disease-modifying agents. Accordingly, the introduction of LPs as a routine diagnostic procedure (and not only for research purposes, see Dubois et al.[18]) for selected cases of MCI patients should be considered in the next revisions of diagnostic guidelines for dementia.

## ACKNOWLEDGMENTS

This study was supported by the EU grants cNEUPRO (contract no. LSHM-CT-2007-037950).
